# Hepatocyte growth factor protects PC12 cells against OGD/R-induced injury by reducing iron

**DOI:** 10.1042/BSR20200287

**Published:** 2020-03-31

**Authors:** Siyue Li, Zhong-Ming Qian, Gaojing Xu, Jie Zheng, Yi Wu

**Affiliations:** 1 Department of Rehabilitation Medicine, Huashan Hospital, Fudan University, Shanghai, China; 2 Institute of Translational and Precision Medicine, Nantong University, Nantong 226001, China

**Keywords:** ferritin light chain (Ft-L), ferroportin 1 (Fpn1), Hepatocyte growth factor (HGF), hepcidin, iron regulatory protein 1(IRP1), neuroprotection, oxygen-glucose deprivation and reoxygenation (OGD/R), PC12 cells, transferrin receptor 1 (TfR1)

## Abstract

In the light of hepatocyte growth factor (HGF) the inhibiting role on the expression of hepcidin, we hypothesized that HGF might be able to reduce cell and tissue iron by increasing ferroportin 1 (Fpn1) content and Fpn1-mediated iron release from cells and tissues. The hypothesized ability of HGF to reduce iron might be one of the mechanisms associated with its neuroprotective action under the conditions of ischemia/reperfusion (I/R). Here, we investigated the effects of HGF on the expression of hepcidin as well as transferrin receptor 1 (TfR1), divalent metal transporter 1 (DMT1), Fpn1, ferritin and iron regulatory proteins (IRPs) in oxygen-glucose deprivation and reoxygenation (OGD/R)-treated PC12 cells by real-time PCR and Western blot analysis. We demonstrated that HGF could completely reverse the OGD/R-induced reduction in Fpn1 and IRP1 expression and increase in ferritin light chain protein and hepcidin mRNA levels in PC12 cells. It was concluded that HGF protects PC12 cells against OGD/R-induced injury mainly by reducing cell iron contents via the up-regulation of Fpn1 and increased Fpn1-mediated iron export from cells. Our findings suggested that HGF may also be able to ameliorate OGD/R or I/R-induced overloading of brain iron by promoting Fpn1 expression.

## Introduction

Hepatocyte growth factor (HGF) is a pleiotropic cytokine [1,2]. The binding of HGF to its unique tyrosine kinase receptor, c-Met, induces activation of Met tyrosine kinase and the autophosphorylation of tyrosine residues in Met [3,4]. The activation of c-Met recruits adaptor molecules and activates several intracellular signaling pathways, and the signaling cascade results in specific cellular programs such as promoting angiogenesis, inhibiting fibrosis and apoptosis, regulating inflammation, and stimulating tissue regeneration [5,6]. HGF and c-Met are both expressed in the developing and adult mammalian brain [7,8].

A number of studies have demonstrated that HGF markedly improves recovery in learning and memory dysfunction and disruption of the blood–brain barrier induced by cerebral ischemia in microsphere-embolized rats [9], attenuates neurological deficits in a rat transient middle cerebral artery occlusion (MCAO) model [10], induces long-term neuroprotection and stroke recovery in male C57/BL6N mice [11], prevents the delayed death of hippocampal neurons in Mongolian gerbils with transient forebrain ischemia [12], protects hippocampal neurons from apoptotic cell death after transient forebrain ischemia in male Wistar rats [13], reduces infarct size and the number of TUNEL-positive cells, promotes the neurogenesis, angiogenesis, and synaptogenesis, and inhibits fibrotic change in brains in rats subjected to transient middle cerebral artery occlusion (tMCAO) [14]. However, the mechanisms involved in the neuroprotective action of HGF have not been completely elucidated.

Iron is the most abundant trace metal in the brain. As in all cells, neurons require iron for many aspects of their physiology [15]. On the other hand, iron is also a major generator of reactive oxygen species (ROS) when it is increased abnormally in the brain. The iron-induced ROS are capable of damaging biological molecules and leading to neuronal injury [16,17]. Additionally, an excess of iron has been considered pathological in the ischemic brain, being a major source of ROS and a contributor to ischemia/reperfusion (I/R)- or hypoxia/reoxygenation (H/R)-induced brain injury [18–22]. Furthermore, HGF has a significant effect on iron homeostasis by suppressing hepcidin [23,24], a central player in body iron metabolism [25].

In the light of these findings, we hypothesized that HGF might be able to reduce brain iron by inhibiting hepcidin expression and then increasing ferroportin 1 (Fpn1) contents and Fpn1-mediated iron release from cells. The hypothesized ability of HGF to reduce iron might be one of the mechanisms associated with its neuroprotective action under the conditions of I/R and H/R. To test this hypothesis, we investigated the effects of HGF on the expression of hepcidin as well as on cell-iron importers transferrin receptor 1 (TfR1) and divalent metal transporter 1 (DMT1), cell-iron exporter Fpn1, cell-iron storage protein ferritin, and iron regulatory protein 1 and 2 (IRP1 and 2) in oxygen-glucose deprivation and reoxygenation (OGD/R)-treated PC12 cells. We demonstrated that HGF protects PC12 cells against OGD/R-induced injury mainly by reducing cell iron contents via the up-regulation of Fpn1 and increased iron export from cells via Fpn1. These findings suggested that HGF may also be able to ameliorate OGD/R-induced overloading of brain iron by promoting the expression of iron export protein Fpn1 *in vivo*.

## Materials and methods

### Chemicals

Unless otherwise stated, all chemicals including mouse monoclonal anti-β-actin were obtained from Sigma-Aldrich Chemical Co., St. Louis, MO, U.S.A. The recombinant mouse HGF protein was obtained from R&D Systems, Minneapolis, MN, U.S.A.; rabbit polyclonal anti-mouse Fpn1 from Novus Biologicals, Littleton, CO, U.S.A.; mouse anti-human TfR1 from Invitrogen Life technologies, Carlsbad, CA, U.S.A.; rabbit polyclonal anti-ferritin light chain (Ft-L) and anti-DMT1 from Proteintech, Chicago, IL, U.S.A.; and rabbit monoclonal anti-IRP1 and anti-IRP2 were from Abcam, San Francisco, CA, U.S.A. Goat anti-rabbit or anti-mouse IRDye 800 CW secondary antibodies were bought from LI-COR Bio Sciences, Lincoln, NE, U.S.A.; the bicinchoninic acid (BCA) protein assay kit and AevertAid First Strand cDNA Synthesis Kit from Thermo Fisher Scientific, Waltham, MA, U.S.A.; the TRIzol reagent from Life technologies, Carlsbad, CA, U.S.A.; FastStart Universal SYBR Green Master and LightCycler96 from Roche, Nutley, NJ, U.S.A.; protein RIPA lysis buffer from Beyotime Institute of Biotechnology, Haimen, JS, China. Recombinant HGF protein was dissolved in phosphate buffer saline (PBS) and diluted to 1–120 ng/ml before use.

### PC12 cell culture

PC12 cells (rat adrenergic neural tumourphaeochromocytoma cell line) were purchased from the Institute of Cell Biology, Chinese Academy of Sciences, Shanghai, China and cultured in DMEM (Thermo Fisher Scientific, Waltham, MA, U.S.A.) supplemented with 10% horse serum, 5% fetal bovine serum,100 units/ml penicillin and 100 mg/ml streptomycin in a 5% CO_2_ incubator (TC2323) at 37°C. The culture medium was changed every other day [26,27].

### Oxygen-glucose deprivation and reoxygenation (OGD/R)

To model ischemic-like conditions *in vitro*, the cells were subjected to OGD, which was achieved by placing cells in DMEM without glucose and in a dedicated chamber (NAPCO 7101FC-1) with 1% O_2_, 94% N_2_ and 5% CO_2_ for 6 h at 37°C as previously described [28,29]. After OGD, cells were incubated with the original medium in a normoxic incubator for 24 h [30]. All experimental protocols were performed according to the Animal Management Rules of the Ministry of Health of China, and approved by the Animal Ethics Committees of Fudan University and Nantong University.

### Cell viability

Cell viability was assessed using an MTT assay as previously described [31,32]. Briefly, a total of 25 ml MTT (1 g/l in PBS) was added to each well before incubation was conducted at 37°C for 4 h. The assay was stopped by the addition of a 100 ml lysis buffer (20% SDS in 50% N'Ndimethylformamide, pH 4.7). Optical density (OD) was measured at the 570 nm wavelength by the use of an ELX-800 microplate assay reader (Bio-tek, Winooski, VT, U.S.A.) and the results were expressed as a percentage of the absorbance measured in the control cells.

### Isolation of total RNA and quantitative real-time PCR

The extraction of total RNA and preparation of cDNA were performed using a TRIzol reagent and the AevertAid FirstStrand cDNA Synthesis Kit respectively, in accordance with the instructions of the manufacturers. Real-time PCR was carried out by RT-PCR instrument (LC96, Roche, Switzerland) using Fast Start Universal SYBR Green Master and the Light Cycler96. The specific pairs of primers used for PCR were: hepcidin forward, 5′-gaaggcaagatggcactaagca-3′; hepcidin reverse, 5′-tctcgtctgttgccggagatag-3′; and β-actin forward, 5′-aaatcgtgcgtgacatcaaaga-3′ and β-actin reverse, 5′-gccatctcctgctcgaagtc-3′. The cycle threshold value of each target gene was normalized to that of the β-actin mRNA [33,34]. Relative gene expression was calculated by the 2^−∆∆Ct^ method [35].

### Western blotting

The cells were washed and homogenized by protein RIPA lysis buffer as described previously [36,37]. Soluble proteins were collected after centrifugation at 13,200 rpm for 15 min at 4°C and protein content was determined using the BCA protein assay reagent kit. Aliquots of the extract containing 30 μg of protein were loaded and run on a single track of 10% SDS–PAGE and then transferred onto a pure nitrocellulose membrane (Bio-Rad, Hercules, CA, U.S.A.). The blots were blocked with 5% non-fat milk and then incubated overnight at 4°C with primary antibodies: mouse monoclonal anti-TfR1 (1:1000), rabbit polyclonal anti-DMT1 (1:1000), rabbit polyclonal anti-Fpn1 (1:1000), rabbit polyclonal anti-Ft-L (1:1000), mouse monoclonal anti-IRP1 (1:1000), anti-IRP-2 (1:1000) overnight at 4°C. After being washed three times, the blots were incubated with goat anti-rabbit (1:5000) or anti-mouse IRDye800 CW secondary antibody (1:5000) for 2 h at room temperature. The intensities of the specific bands were detected and analyzed by the Odyssey infrared imaging system (Li-Cor, Lincoln, NE, U.S.A.). To ensure even loading of the samples, the same membrane was probed with a mouse monoclonal anti-β-actin antibody (1:5000) as internal protein controls.

### Statistical analysis

Statistical analyses were performed using Graphpad Prism 8.0. Data are presented as mean ± SEM. Differences between the means were determined through *t*-tests or one-way analysis of variance. A *P* < 0.05 denoted statistical significance.

## Results

### HGF protected PC12 cells from OGD/R-induced injury

**Figure 1:**
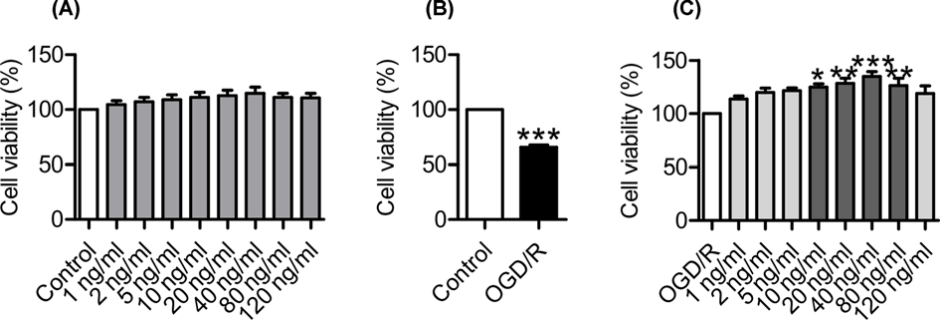
HGF protected PC12 cells from OGD/R-induced injury PC12 cells were pretreated with 0 (Control), 1, 2, 5, 10, 20, 40, 80 or 120 ng/ml of HGF for 1 h and then exposed to normoxia for 30 h (**A**), 0 ng/ml of HGF for 1 h and then exposed to normoxia for 30 h (Control) or OGD (oxygen-glucose deprivation) for 6 h / R (reoxygenation) for 24 h (OGD/R) (**B**), 0 (OGD/R), 1, 2, 5, 10, 20, 40, 80 or 120 ng/ml of HGF for 1 h and then exposed to OGD for 6 h / R for 24 h (**C**). Cell viability was then measured as described in ‘Materials and Methods’ section. Data were represented as mean ± SEM (*n*=4). ***P<0.001 vs. the control in (B), or OGD/R in C; **P<0.01 vs. OGD/R in (C).

We first investigated the effects of HGF on cell viability by pre-treatment of PC12 cells with different concentrations (0, 1, 2, 5, 10, 20, 40, 80 and 120 ng/ml) of HGF for 1 h before exposing the cells to normoxia for 30 h. There were no significant differences in cell viabilities between the cells treated with varying concentrations of HGF and the controls (Figure 1A), demonstrating that HGF had no significant effect on cell viability under normoxic conditions. Then, we examined the effects of HGF on cell viability under OGD/R conditions by pre-treatment of PC12 cells with different concentrations (0–120 ng/ml) of HGF before exposing the cells to OGD for 6 h / R (reoxygenation) for 24 h. It was found that viability in the cells treated with 0 ng/ml of HGF plus OGD/R was significantly lower than that in the control cells (0 ng/ml HGF plus normoxia) (Figure 1B), implying that OGD/R could induce a significant cell-injury under our *in vitro* experimental conditions. However, the viability in the cells treated with 10, 20 40 or 80 ng/ml of HGF was found to be significantly higher than that in the cells treated with 0 ng/ml of HGF plus OGD/R (Figure 1C), showing that HGF could protect PC12 cells from OGD/R-induced injury. The maximum protective effect was found at 40 ng/ml, and this dosage was therefore selected to be used in the following experiments.

### HGF up-regulated Fpn1, down-regulated Ft-L and had no significant effect on TfR1 and DMT1 expression in OGD/R-treated PC 12 cells

**Figure 2:**
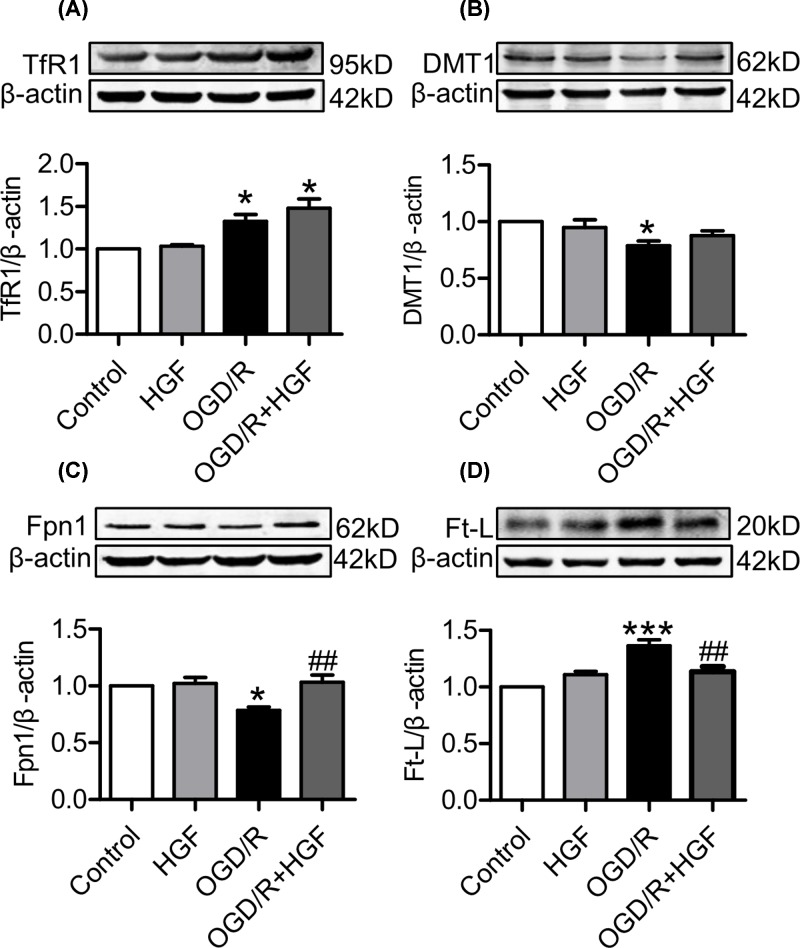
HGF up-regulated Fpn1, down-regulated Ft-L and had no significant effect on TfR1 and DMT1 expression in OGD/R-treated PC 12 cells PC12 cells were pretreated with 0 (Control) or 40 ng/ml of HGF for 1 h and then exposed to normoxia for 30 h or OGD for 6 h / R for 24 h. Expression of TfR1 (**A**), DMT1 (**B**), Fpn1 (**C**) and Ft-L (**D**) was detected by Western blot analysis as described in ‘Materials and Methods’ section. Data were represented as mean ± SEM (*n*=4). *P<0.05, ***P<0.001 vs. the control; ##*P*<0.01 vs. the OGD/R-treated group.

To find out the potential mechanisms involved in the protection of HGF on PC12 cells from OGD/R-induced injury, we investigated the effects of HGF on iron-uptake proteins TfR1 and DMT1, iron-release protein Fpn1 and iron-storage protein Ft-L in OGD/R-treated PC 12 cells by pretreatment of PC12 cells with 0 or 40 ng/ml of HGF and then exposure to normoxia or OGD/R. Expression of TfR1 (Figure 2A), DMT1 (Figure 2B), Fpn1 (Figure 2C) and Ft-L (Figure 2D) in the cells treated with different concentrations of HGF was found to have no significant difference from that in the control cells under normoxic conditions. However, treatment with OGD/R induced a significant increase in the expression of TfR1 and Ft-L and a reduction in DMT1 and Fpn1. The expression of TfR1 (Figure 2A) and DMT1 in the cells co-treated with 40 ng/ml of HGF+OGD/R was higher than that in the cells treated with OGD/R only, but the difference was not significant. The expression of Fpn1 in the cells co-treated with HGF+OGD/R was shown to be significantly lower than that in the cells treated with OGD/R only and showed no difference from that in the cells treated by 40 ng/ml of HGF only (Figure 2C), indicating that HGF could completely restore the effect of OGD/R on Fpn1. The content of Ft-L in the cells co-treated with HGF+OGD/R was significantly lower than that in the cells treated with OGD/R only, showing that HGF was able to decrease intracellular iron storage in the OGD/R-treated PC12 cells (Figure 2D).

### HGF down-regulated hepcidin mRNA and up-regulated IRP1 protein expression in OGD/R-treated PC12 cells

**Figure 3:**
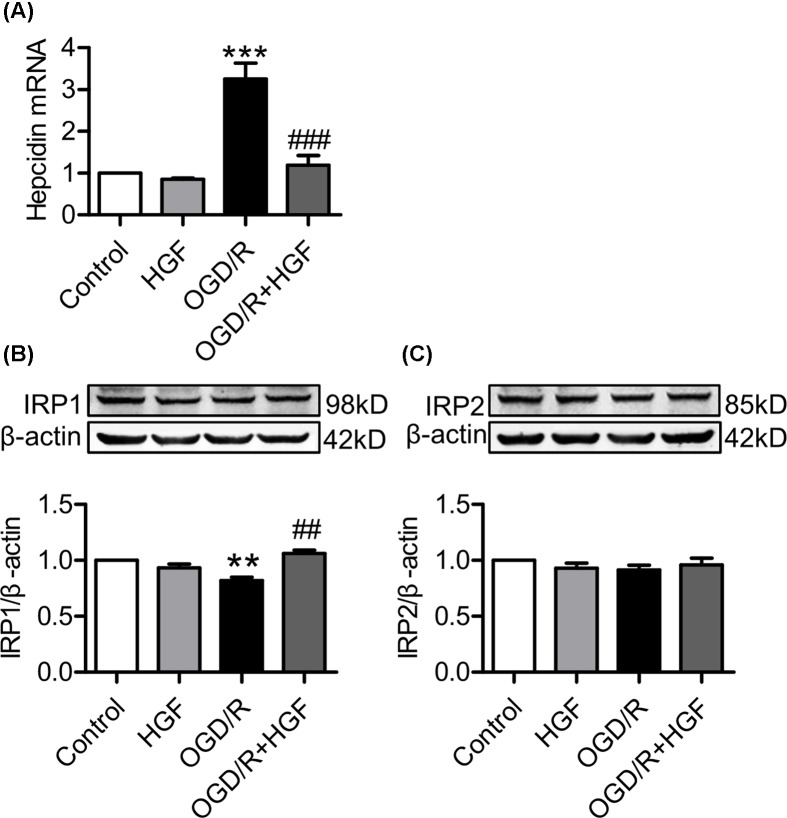
HGF down-regulated hepcidin mRNA and up-regulated IRP1 protein expression in OGD/R-treated PC12 cells PC12 cells were pretreated with 0 (Control) or 40 ng/ml of HGF for 1 h and then exposed to normoxia for 30 h or OGD for 6 h / R for 24 h. Expression of hepcidin mRNA (**A**), IRP1 (**B**) and IRP2 proteins (**C**) were detected by RT-PCR or Western blot analysis as described in ‘Materials and Methods’ section. Data were represented as mean ± SEM (A: *n*=3; B and C: *n*=4). **P<0.01, ***P<0.001 vs. Control; ##*P*<0.01, ^###^*P*<0.001 vs. OGD/R.

It has been well-documented that mammalian iron metabolism or the expression of iron transport and storage proteins is regulated cellular by IRPs and systemically by hepcidin [38,39]. To understand how HGF affects the expression of iron transport and storage proteins, we subsequently investigated the effects of HGF on the expression of IRP protein and hepcidin mRNA in OGD/R-treated PC12 cells by pretreatment with 0 or 40 ng/ml of HGF and then exposure to normoxia or OGD/R. Same as the effects of HGF on cell-iron transport and storage proteins, treatment with HGF did not induce any significant effect on the expression of hepcidin mRNA (Figure 3A) and IRP1 (Figure 3B) and IRP2 (Figure 3C) proteins under normoxic conditions. Also, there were no significant differences in the expression of IRP2 between the cells co-treated with HGF+OGD/R and the cells treated with OGD/R only or the cells treated HGF only, indicating that HGF had no effect on IRP2 expression in the OGD/R-treated cells. However, treatment with OGD/R induced a significant increase in the expression of hepcidin mRNA and reduction in IRP1 protein, and these effects of OGD/R could be markedly reversed by co-treatment with HGF. The expression of hepcidin mRNA was significantly lower (Figure 3A) and IRP1 protein higher (Figure 3B) in the cells co-treated with HGF+OGD/R than that in the cells treated with OGD/R only, no different from that in the cells treated with HGF only, implying that HGF could completely abolish the effect of OGD/R on hepcidin and IRP1.

## Discussion

Cell iron content or balance is mainly dependent on the expression of iron uptake and release proteins, TfR1, DMT1 and Fpn1.TfR1 and DMT1 are key proteins of iron uptake and the TfR1/DMT1-mediated transport of transferrin-bound iron (Tf-Fe) is the main route for mammalian cellular iron accumulation [40]. Fpn1 is the only identified cellular iron exporter currently [25]. Ferritin has two isoforms; the heavy chain (Ft-H) and Ft-L, Ft-H has a ferroxidase activity [41], while Ft-L lacks detectable ferroxidase activity but can store more iron [42], being a good marker of cell iron content. In the present study, we demonstrated that the content of Ft-L in the cells co-treated with HGF+OGD/R was significantly lower than that in the cells treated with OGD/R only, indicating that that HGF is able to reduce intracellular iron storage in the OGD/R-treated PC12 cells. Meanwhile, we found that HGF up-regulates Fpn1 and had no significant effect on TfR1 and DMT1 expression in OGD/R-treated PC 12 cells, providing evidence that the ability of HGF to reduce cell iron contents was mainly due to its role to up-regulate Fpn1 and to increase Fpn1-mediated iron export from cells.

Mammalian iron metabolism is regulated systemically by hepcidin [38,39]. Hepcidin is a central regulator of systemic iron homeostasis [30,43–46]. Fpn1 (IREG1/Slc40a1/MTP1) is a membrane receptor of hepcidin and the binding of hepcidin with Fpn1 induces the internalization and degradation of the Hepcidin/Fpn1 complex, reducing the number of Fpn1 on the membrane [47]. To find out how HGF affects the expression of Fpn1, we investigated the effects of HGF on the expression of hepcidin mRNA. We demonstrated that HGF down-regulated hepcidin mRNA in OGD/R-treated PC12 cells. The finding was consistent with what has been reported by others [23,24] and also implied that the increased expression of Fpn1 was mainly due to the role of HGF to down-regulate the expression of hepcidin, namely that the HGF-induced up-regulation of Fpn1 expression was a hepcidin-mediated process.

In addition to hepcidin, the expression of TfR1, DMT1, Fpn1 and ferritin is also co-ordinately regulated by the IRP/IRE (iron-responsive element) system at cellular level [38,39,48,49]. The binding of IRPs may stabilize genes with IREs on 3′ untranslated regions (UTRs) (TfR1 and DMT1) and suppress the translation of genes with IREs on 5′ UTR (Fpn1 and Ferritin) [50]. The increased IRP1 could therefore induce an increase in TfR1 and DMT1 expression as well as a reduction in Fpn1 and ferritin expression. In the present study, we also examined the effects of HGF on the expression of IRP1 and IRP2 proteins and found that IRP1, but not IRP2, was significantly increased by treatment with HGF in OGD/R-treated PC12 cells. This might be associated with the increased expression of TfR1 and DMT1 (although this was not found to be statistically significant) in the cells co-treated with HGF+OGD/R as compared with the cells treated with OGD/R only. The finding may also imply that HGF-induced reduction in Ft-L content was due to not only the changes in hepcidin and Fpn1 but also in increased IRP1, and also suggests that hepcidin might play a dominant role, as compared with IRP/IRE system, in the control of Fpn1 expression in OGD/R-treated PC12 cells.

**Figure 4:**
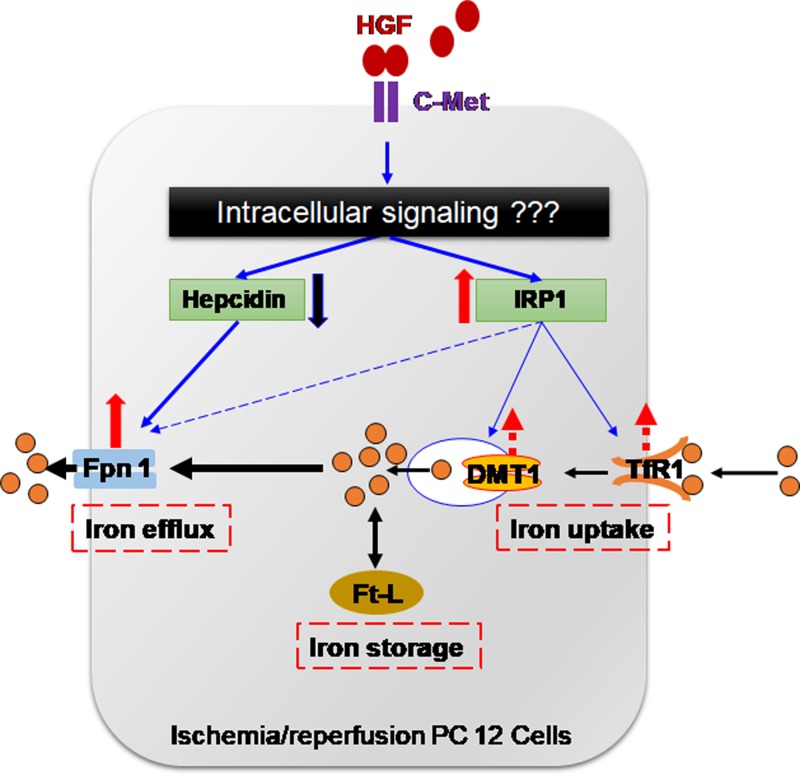
A hypothetical scheme for hepatocyte growth factor (HGF) to affect the expression of iron transport, storage and regulatory proteins or mRNA in PC12 cells treated with oxygen-glucose deprivation/reoxygenation (OGD/R) The binding of HGF with its receptor (c-Met) activates intracellular signaling pathways. The activated signaling pathways induce a significant reduction in hepcidin and an increase in IRP1 expression. Fpn1 expression is controlled by hepcidin as well as IRP; however, hepcidin may play a dominant role under the conditions of OGD/R. Therefore, the increased expression of Fpn1 is mainly due to the down-regulation of the hepcidin expression induced by HGF. The increased Fpn1 expression and Fpn1-mediated iron release from the cells leads to a significant reduction in Ft-L (cell iron) content. The slight increase in TfR1 and DMT1 expression may be due to the increased expression of IRP1. The intracellular signaling pathways activated by the binding of HGF with the c-Met receptor are unknown, and the further studies about this issue are needed. (c-Met: Receptor for hepatocyte growth factor; DMT1: divalent metal transporter 1; Fpn1: ferroportin 1; Ft-L: ferritin-light chain; HGF: hepatocyte growth factor; IRP1: iron regulatory protein 1; TfR1: transferrin receptor 1). Solid- arrows or lines: Significant; Dotted-arrow or lines: Not significant.

In conclusion, we demonstrated for the first time that HGF was able to completely reverse the OGD/R-induced reduction in the expression of Fpn1 and IRP1 proteins and increase in the contents of Ft-L protein and hepcidin mRNA in PC12 cells. These findings provided evidence supporting our hypothesis and showed that HGF was able to reduce brain iron by inhibiting hepcidin expression and then increasing ferroportin 1 (Fpn1) contents and Fpn1-mediated iron release from the cells (Figure 4). These findings suggest that HGF may also be able to ameliorate OGD/R-induced overloading of brain iron by promoting the expression of iron export protein Fpn1 *in vivo*. The intracellular signaling pathways, activated by the binding of HGF with the c-Met receptor and involved in the regulation of HGF on hepcidin and IRP expression (Figure 4), are unknown. Further studies about this issue are needed.

## Abbreviations

AKT: protein kinase B
DMT1: divalent metal-ion transporter 1
Fpn1: ferroportin 1
Ft-L: ferritin light chain
HGF: hepatocyte growth factor
IRE: iron response element
IRP1: iron regulatory protein 1
Met: c-Mettyrosine-protein kinase (hepatocyte growth factor receptor)
OGD/R: oxygenglucose deprivation/reoxygenation
PI3K: phosphatidylinositol 3-kinase
TfR1: transferrin receptor 1
